# Tumor core biopsies adequately represent immune microenvironment of high-grade serous carcinoma

**DOI:** 10.1038/s41598-019-53872-1

**Published:** 2019-11-26

**Authors:** Olivia D. Lara, Santhoshi Krishnan, Zhihui Wang, Sara Corvigno, YanPing Zhong, Yasmin Lyons, Robert Dood, Wei Hu, Lisha Qi, Jinsong Liu, Robert L. Coleman, Shannon N. Westin, Nicole D. Fleming, Vittorio Cristini, Arvind Rao, Jared Burks, Anil K. Sood

**Affiliations:** 1 Department of Gynecologic Oncology and Reproductive Medicine, The University of Texas MD Anderson Cancer Center, Houston, TX 77030 USA; 2 Department of Electrical and Computer Engineering, Rice University, Houston, TX 77030 USA; 3 Department of Computational Medicine and Bioinformatics, University of Michigan, Ann Arbor, MI 48109 USA; 4 Mathematics in Medicine Program, Houston Methodist Research Institute, Houston, TX 77030 USA; 5 Department of Pathology, The University of Texas MD Anderson Cancer Center, Houston, TX 77030 USA; 6 Department of Pathology, The First Hospital of Jilin University, Changchun, China; 7 Department of Imaging Physics, The University of Texas MD Anderson Cancer Center, Houston, TX 77030 USA; 8 Department of Radiation Oncology, University of Michigan, Ann Arbor, MI 48109 USA; 9 Flow Cytometry and Cell Imaging, The University of Texas MD Anderson Cancer Center, Houston, TX 77030 USA; 10 Center for RNA Interference and Non-Coding RNA, The University of Texas MD Anderson Cancer Center, Houston, TX 77030 USA

**Keywords:** Cancer microenvironment, Tumour heterogeneity, Translational research

## Abstract

The prognostic and therapeutic value of the tumor microenvironment (TME) in various cancer types is of major interest. Characterization of the TME often relies on a small representative tissue sample. However, the adequacy of such a sample for assessing components of the TME is not yet known. Here, we used immunohistochemical (IHC) staining and 7-color multiplex staining to evaluate CD8 (cluster of differentiation 8), CD68, PD-L1 (programmed death-ligand 1), CD34, FAP (fibroblast activation protein), and cytokeratin in 220 tissue cores from 26 high-grade serous ovarian cancer samples. Comparisons were drawn between a larger tumor specimen and smaller core biopsies based on number and location (central tumor vs. peripheral tumor) of biopsies. Our analysis found that the correlation between marker-specific cell subsets in larger tumor *versus* smaller core was stronger with two core biopsies and was not further strengthened with additional biopsies. Moreover, this correlation was consistently strong regardless of whether the biopsy was taken at the center or at the periphery of the original tumor sample. These findings could have a substantial impact on longitudinal assessment for detection of biomarkers in clinical trials.

## Introduction

The tumor microenvironment (TME) is a complex network of interactions between immune cell populations, cancer cells, and vascular and stromal components, which play a critical role in cancer cell growth and progression. It is now well recognized that many components of the TME have implications for patient outcome and therapeutic targeting^1^. For example, the presence of T-cell infiltration has been reported to consistently correlate with improved patient survival^2–4^, whereas increased expression of tumor-associated macrophages has been associated with poor clinical outcome^5–7^.

Many approaches to assess the TME have been examined, including biological and computational models based on bulk tumor or single-cell technology^8–11^, but these have been limited by the lack of adequate tissue sample and cost. Assessing the adequacy of a tissue sample for TME investigations is a central issue. Numerous therapies targeting the TME have emerged, many of which have relied on serial biopsies to assess the longitudinal effect of therapy on the TME over time. Clinically, serial tumor biopsies have been applied to evaluate putative predictive biomarkers and to test for target-specific effects with novel therapies^12^. However, it is unclear whether serial biopsies adequately represent the heterogeneity of tumor specimens^13^.

Here, we used immunohistochemical (IHC) staining and advanced staining techniques to probe high-grade serous ovarian carcinoma (HGSC) samples to quantify immune and stromal cell populations. Furthermore, we determined the variability between cores and the ideal number of cores based on the location needed to adequately assess the tumor immune environment. We found that TME components can be assessed reliably with a minimum of three small tissue biopsies taken at random locations within the larger tumor.

## Materials and Methods

### Patient cohorts

Our study population consisted of 26 chemotherapy-naive patients with HGSC. Formalin-fixed, paraffin-embedded (FFPE) tissue blocks acquired at surgery were derived from primary or metastatic tumor sites. A tissue microarray (TMA) was assembled with 1.5-mm cores punched from representative areas. For 10 patients, 5 tissue cores were taken at random locations within the tumor; for 5 patients, 10 tissue cores were taken randomly; and for 12 patients, 10 tissue cores were taken, 5 of which were taken from central tumor and 5 from peripheral tumor regions. Three TMA blocks were assembled with a total of 220 cores. All methods were performed in accordance with the relevant guidelines and regulations of The University of Texas MD Anderson Cancer Center and were approved by its ethical committee and Institutional Review Board. Written informed consent was obtained concerning the analysis of tumor tissue for scientific purposes.

### IHC and 7-color multiplex staining

Slides were cut from TMAs obtained from tumor blocks and from original tumor blocks, deparaffinized, rehydrated, and underwent IHC analysis. Initial IHC analyses consisted of staining with CD8 (cluster of differentiation 8) and CD68 (cluster of differentiation 68) antibodies and hematoxylin counterstaining (Sigma-Aldrich, GHS316-500). These analyses did not provide data on CD8 or CD68, for which we relied on multiplex staining described below, but allowed us to define tissue segmentation for stroma, epithelial, and blank areas. On average, 10% of cores were lost after processing and staining. Additional sections of FFPE block and TMA were used for 7-color multiplex staining. An Opal 7-color manual IHC kit (PerkinElmer, NEL 811001KT, lot # 2398178) was used for sequential staining (see Supplementary Fig. S1). The protocol was based on the use of fluorescent tyramide signal amplification (TSA) reagents that retain a fluorescence signal after multiple treatments with a steamer to remove the primary and secondary antibodies. Primary antibody concentration and brands are included in Supplementary Table S1. After sequential reactions, slides were counterstained with DAPI (PerkinElmer, FP1490) and mounted with Prolong Antifade fluorescence mounting medium (Invitrogen, P36965). Single marker staining was used to compose a spectral library, which was analyzed in order to set the exposure times to detect specific signals. Data were exported as.txt files.

### Imaging and spectral unmixing

IHC and multiplex-stained slides were imaged with use of the Vectra Multispectral Imaging system version 2 (PerkinElmer). All samples were scanned at 20× magnification for TMA annotation and larger tumor region selection. Low-powered images were then used to extract one 40× image of each TMA core and 100 to 300 images of the larger patient samples, depending on the size of the samples, with the use of a Phenochart slide viewer (see Supplementary Fig. S2). Filter cubes used for multispectral imaging were DAPI (440–680 nm), FITC (520–680 nm), Cy3 (570–690 nm), Texas Red (580–700 nm), and Cy5 (670–720 nm). The signal intensities for each marker were normalized, and spectral unmixing was performed with PerkinElmer inForm Analysis software (2.4.1.). An image encompassing the entire slide through the full emission spectrum of each filter (DAPI, fluorescein isothiocyante [FITC]), Cy3, Texas Red [TR], and Cy5) was captured. A spectral signature for each fluorophore was obtained by using the same multispectral imaging protocol of single-stained slides, as well as an unstained slide to obtain the auto-fluorescence signature of the tissue. Spectral unmixing was then used to separate these spectral signatures into individual signals (see Supplementary Fig. S3).

### Tissue and cell segmentation

We then used the inForm 2.4.1 image analysis train-by-example interface to develop an algorithm for tissue segmentation and cellular segmentation. Tissues stained with single IHC stain were manually segmented in stromal areas, tumor epithelial areas, and blank areas on a set of 15 training images. Once an accurate algorithm was developed for tissue segmentation, batch processing of all images could be performed. Percentages of tumor and stroma were determined for each core and larger specimen. Cell segmentation was performed on multiplex stained slides by using nuclear definition to draw cell contours, and the train-by-example interface was then used to identify cells on a cell-by-cell basis (see Supplementary Fig. S4). After cellular segmentation, cell phenotyping was performed. Spectral unmixing was used to separate *brown* nuclear [virtual DAB (3,3’-diaminobenzidine)] staining from *blue* (virtual hematoxylin) staining. InForm software was then used to convert the images to quantitative optical density (OD) values. The OD threshold was set to identify positive-staining cells: CD8 (10.00), CD68 (1.84), PD-L1 (0.54), CD34 (3.00), FAP (0.2), and cytokeratin (0.58). Once the algorithm was proven to be reliable, all slides were segmented, reviewed, merged, and exported for analysis. The percentages of tumor and stroma were determined for each core and larger specimen. The percentages of positively stained cells were similarly determined. Data were exported as.txt files.

### Statistical analyses

All of the correlations between the larger tumor specimen and the tumor cores (combined or separately) were identified by using Pearson correlation analysis. A 95% confidence interval (CI) was assumed for the correlation coefficient distributions in all cases. R-values and *P-*values were reported for all comparisons. Strong correlations were based on an R > 0.7, and moderate correlations were based on R-values between 0.5 and 0.7^14^. The percentage of positively stained cell type was used as the comparative feature for the statistical analyses. The means of the correlation distributions were compared by using the Wilcoxon RankSum test, with the null hypothesis that both the samples were selected from the same population distribution. ICC estimates and their 95% confidence intervals were calculated using the ‘irr’ package in R(R Core Team(2018), Vienna, Austria) with a single rating(k = 2), absolute-agreement, 2-way mixed-effects model^15^. The P-value was reported, with significance at P < 0.05. The variance across the TMA and larger slides for each phenotype was compared by using the Levene’s test, with the null hypothesis that the variances of the sample populations are equal. The F-statistic and the P-value were reported, with significance at P < 0.05. All calculations were done on MATLAB 2018b (The MathWorks, Inc., Natick, Massachusetts), and plotting was done with use of both MATLAB and GraphPad Prism 7, version 7.03.

## Results

### Correlation between tumor epithelial and stromal areas

We first used IHC staining to assess tumor epithelial and stromal areas. Our initial analysis focused on the percentage of stromal and epithelial tumor regions among all 26 patient samples. Each patient sample had between 1 and 10 cores available for analysis; these were taken from random, central, and peripheral locations. We compared the percentages of tumor epithelial and stromal areas on the total tumor areas throughout the cores and then separated them into central and peripheral cores. We observed a non-significant correlation (R = 0.15, *P* = 0.48) between stroma in larger tumor samples when compared with cores and similarly a non-significant correlation (R = 0.43, *P* = 0.26) for tumor regions in larger samples versus cores (see Supplementary Fig. 5).

**Figure 1:**
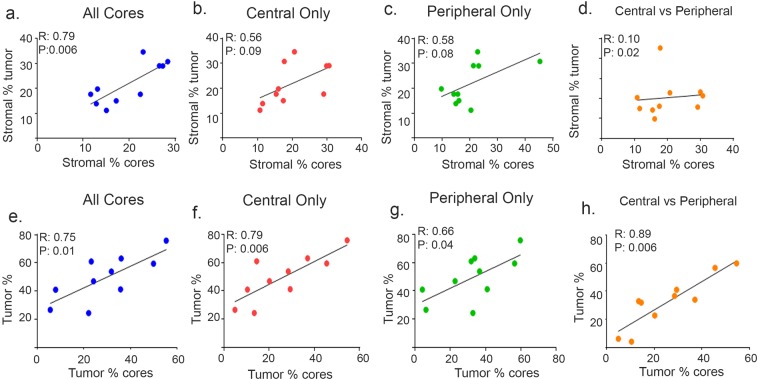
Correlation between stromal and tumor epithelial areas in patients. Stromal correlation computed for larger tumor specimens versus core biopsies for all core biopsies (**a**), central core biopsies (**b**), peripheral core biopsies (**c**), and central versus peripheral core biopsies (**d**). Tumor percentage for larger tumor specimen versus core biopsies for all core biopsies (**e**), central core biopsies (**f**), peripheral core biopsies (**g**), and central versus peripheral core biopsies (**h**). Pearson correlation coefficient (*R*) and *P* values for each correlation analysis are shown.

Next, we repeated our analysis using 11 patient samples that had at least four central cores and four peripheral cores that were available for comparison. We found a strong correlation (R = 0.79, *P* = 0.006) between the stroma percentage in core biopsies and in larger tumor specimens, and this correlation persisted regardless of the location of the core biopsies (central cores, R = 0.56, *P* = 0.09; peripheral cores, R = 0.58, *P* = 0.08) (Fig. 1a,c); the relationship between the cores taken from central *versus* peripheral tumor showed a weak correlation (R = 0.10, *P* = 0.02) (Fig. 1d). The correlation between the larger tumor and all of the cores combined (Fig. 1a) was significant at a 0.05 confidence level. While all of the other correlations (Fig. 1b,c) were identified as non-significant at the 0.05 confidence level, but were significant at the 0.1 confidence level.

Similarly, we observed a strong correlation (R = 0.75, *P* = 0.01) between the percentage of tumor epithelial areas compared with all cores, regardless of the location from which the cores were taken (central cores, R = 0.79, *P* = 0.006; peripheral cores, R = 0.66, *P* = 0.04) (Fig. 1e–g). We then compared central with peripheral cores and found a strong correlation between the two data sets (R = 0.89, *P* = 0.006) (Fig. 1h). Collectively, these results indicate that the percentage of tumor and stroma may be reliably represented by core biopsies, regardless of location. Nevertheless, stroma percentages are more dependent on core location when compared with tumor epithelial area percentages, which seem to be concordantly distributed in peripheral and central cores. We next sought to determine whether the distribution and abundance of different TME cell populations could be accurately detected and measured by analyzing tumor core biopsies with a 7-color multiplex staining.

### Analyses of abundance and distribution of TME populations

**Figure 2:**
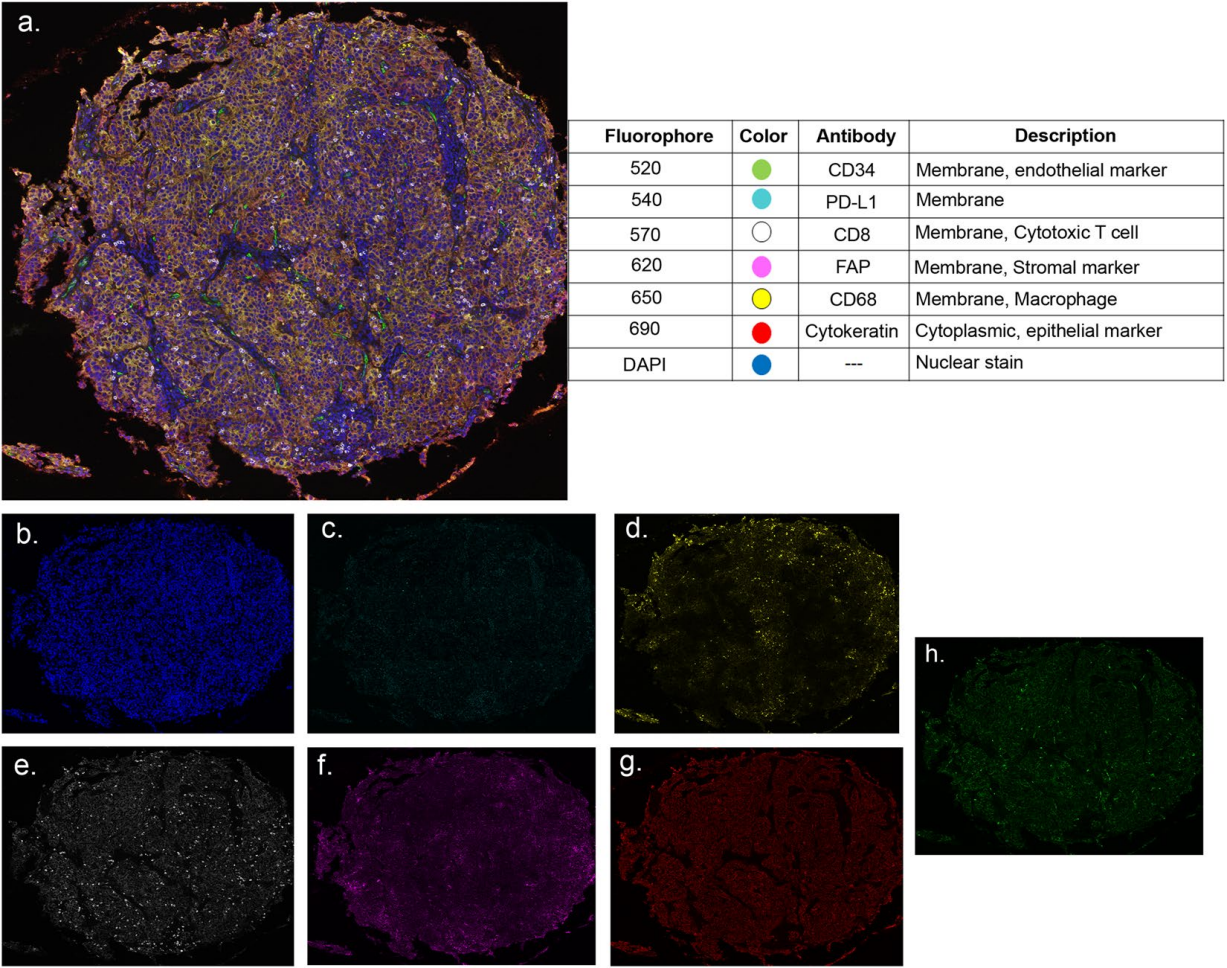
Opal 7-color multiplex analysis. (**a**) Representative images displaying the same TMA core after multispectral imaging and after spectral unmixing; (**b**) nuclear marker DAPI (pseudocolored blue); (**c**) PD-L1 (membrane, 540, pseudocolored cyan); (**d**) CD68 (membrane, 650, pseudocolored yellow); (**e**) CD8 (membrane, 570, pseudocolored white); (**f**) fibroblast-activated protein (membrane, 620, pseudocolored magenta); (**g**) cytokeratin (cytoplasmic, 690, pseudocolored orange); (**h**) CD34 (membrane, 520, pseudocolored green); and autofluorescence (pseudocolored black) not pictured. Inset summary of each defined fluorophore, color code, and associated marker.

**Figure 3:**
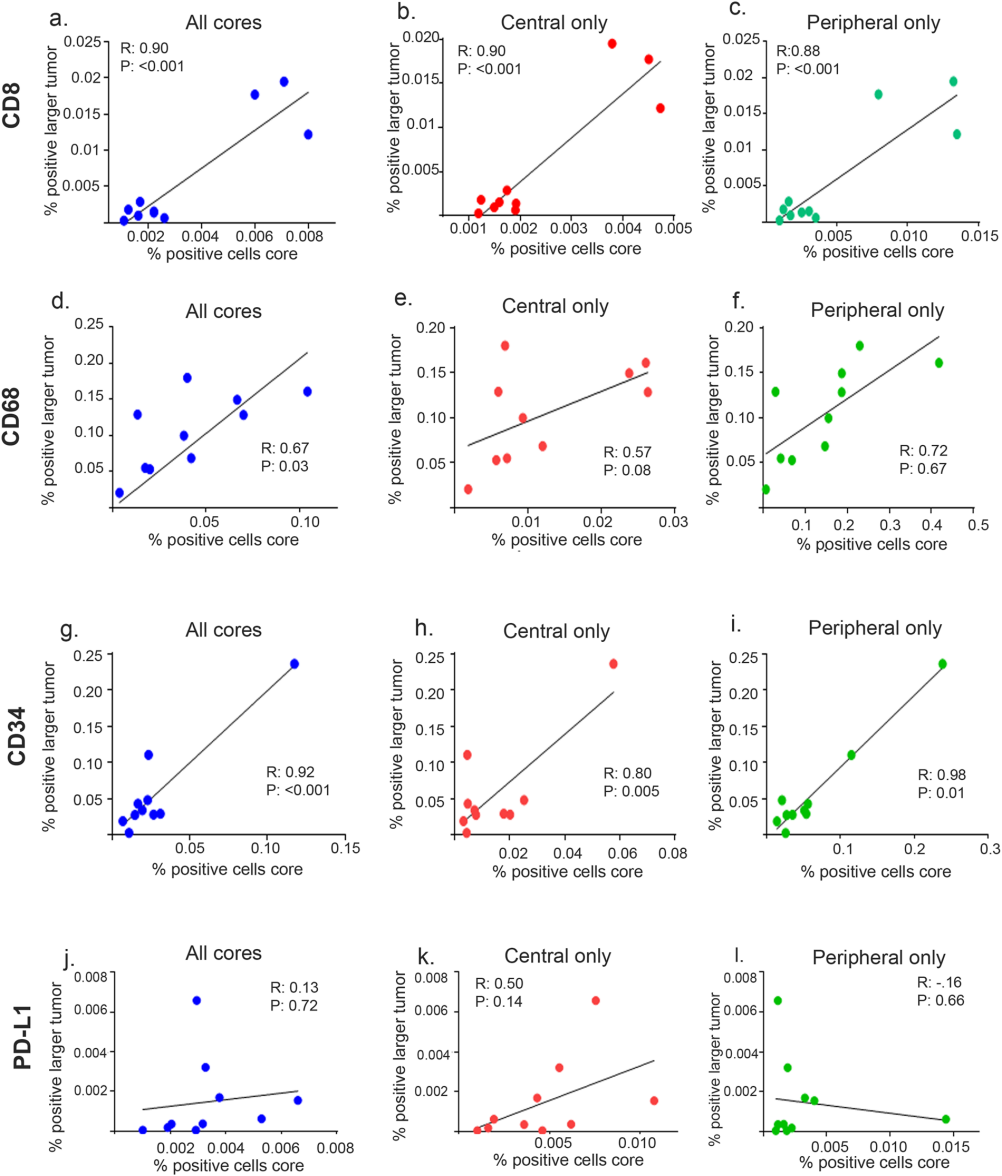
Correlation between marker positive cell counts in large samples versus core biopsies. Correlation between number of positive-stained cells for CD8 (**a**–**c**), CD68 (**d**–**f**), CD34 (**g**–**i**), and PD-L1 (**j**–**l**) in large tissue versus cores. CD8+ correlations between large tumor samples and (**a**) all core biopsies, (**b**) central core biopsies only, and (**c**) peripheral core biopsies only. CD68+ correlation for (**d**) all core biopsies, (**e**) central core biopsies only, and (**f**) peripheral core biopsies only. CD34+ correlations for (**g**) all core biopsies, (**h**) central core biopsies only, and (**i**) peripheral core biopsies only. PD-L1+ correlations for (**j**) all core biopsies, (**k**) central core biopsies only, and (**l**) peripheral core biopsies only. Pearson correlation coefficient (*R*) and *P* values for each correlation analysis are shown in insets.

Next, we used a 7-color multiplex staining by using an Opal multiplex staining protocol, which allowed for simultaneous evaluation of seven markers in a single tissue section (see Supplementary Fig. S1). Multispectral imaging was applied to stained tissue samples (Fig. 2). We observed a strong correlation (all cores, R = 0.90, *P* < 0.001; central cores, R = 0.90, *P* < 0.001; peripheral cores, R = 0.88, *P* < 0.001) between CD8 positively stained cells for the entire specimen compared with all cores, regardless of where the cores were taken (Fig. 3a–c). Similarly, we observed a moderate correlation (R = 0.67, *P* = 0.03) between macrophage (CD68+) populations (Fig. 3d) and a strong correlation (R = 0.92, *P* < 0.001) between cells positive for vessel marker CD34 (Fig. 3g) when comparing the larger tumor specimen and core biopsy. When correlation of larger tumor samples versus central and peripheral tumor biopsies were separately analyzed for macrophage marker CD68, their correlation was not statistically significant (Fig. 3e,f). However the strong correlation persisted in both central and peripheral tumor biopsies (central cores, R = 0.80, *P* = 0.005; peripheral cores R = 0.98, *P* = 0.01) for CD34-positive cells (Fig. 3h,i). Finally, we observed no correlation (R = 0.13, *P* = 0.72) between PD-L1–positively stained cells when all cores were compared (Fig. 3j). When cores were separated into those taken from central versus peripheral tumor, we observed no correlation (Fig. 3k–l).

We then compared central versus peripheral cores for all markers. We observed a strong correlation between central cores and peripheral cores for markers CD8 (R = 0.92, *P* < 0.001), CD68 (R = 0.74, *P* = 0.01), and CD34 (R = 0.76, *P* = 0.01) (see Supplementary Fig. S6), indicating that the location of the core did not affect overall correlation. These results demonstrate that components of the TME, in particular lymphocytes, macrophages, and vessel number, could reliably be represented on core biopsies regardless of location. However, the number of cells that stained PD-L1–positive was less reliably detected on core biopsies in general and also dependent on tumor area location.

### Analysis of the optimal representative number of cores

**Figure 4:**
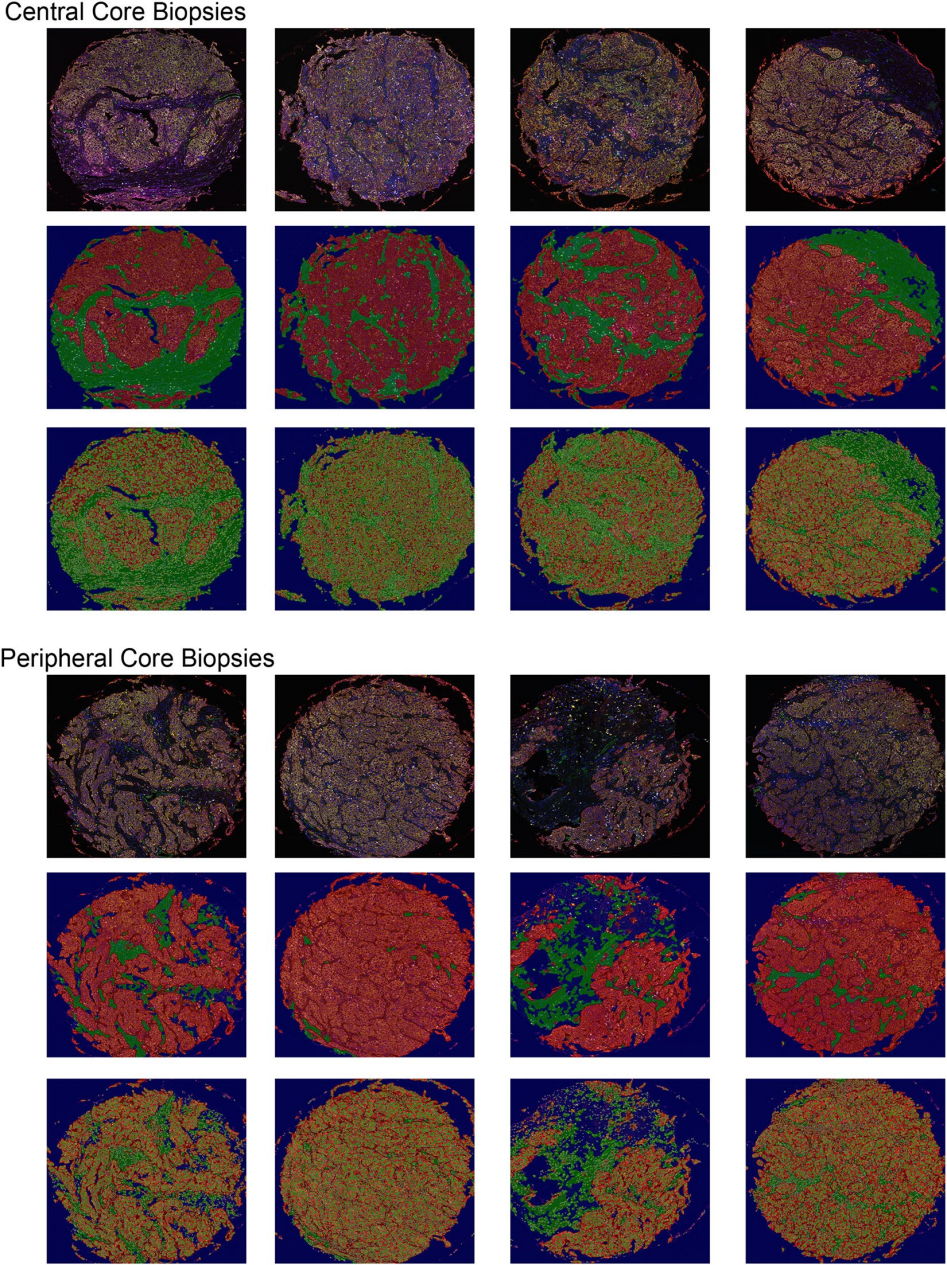
Variability between core specimens. Eight representative core biopsies taken from a single tumor specimen. The upper panel represents biopsies taken from central tumor; the lower panel corresponds to biopsies taken from peripheral tumor. Tissue and cellular segmentation performed on each core biopsies allows for visual representation of variability between cores depending on location taken.

**Figure 5:**
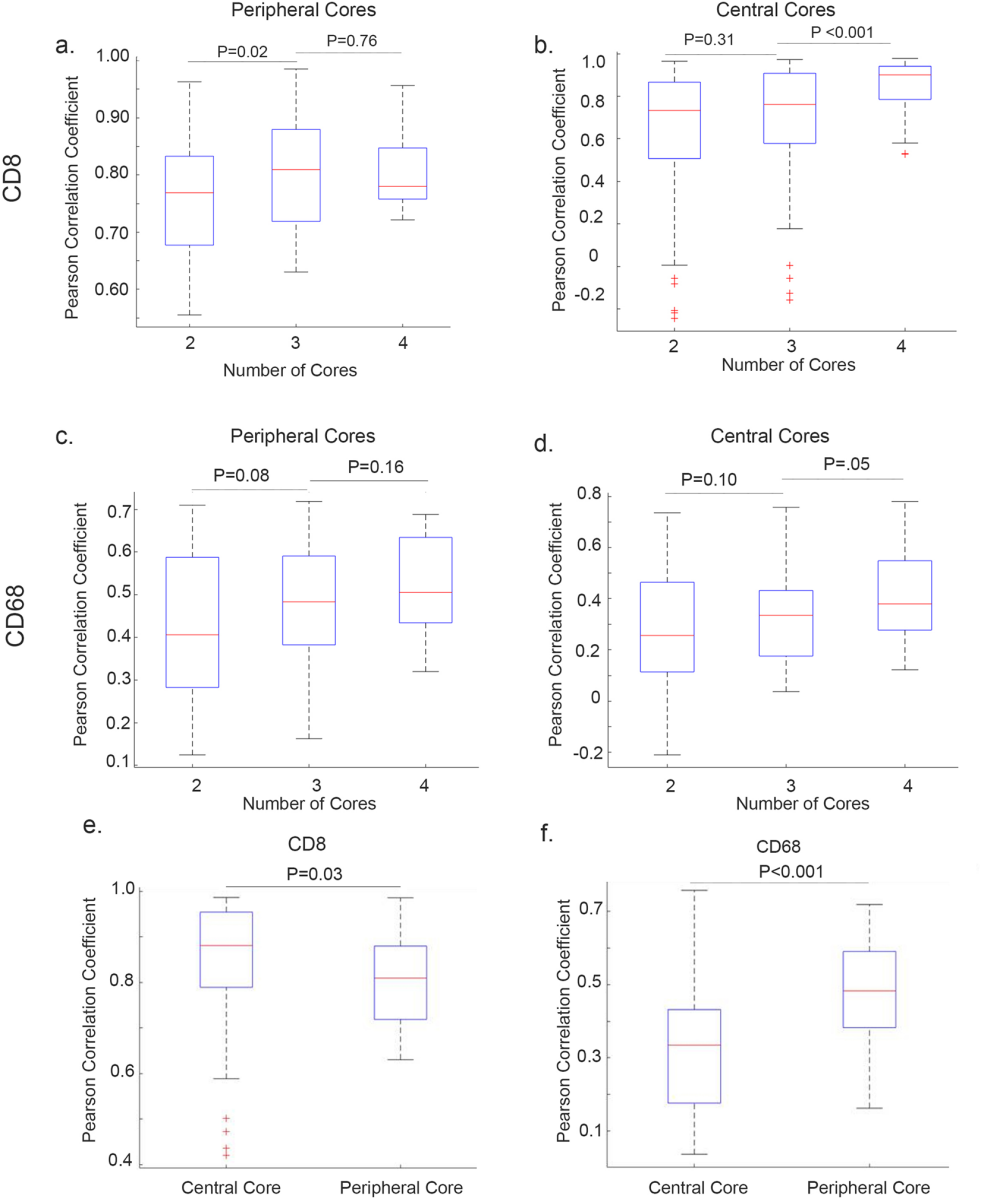
Variability between central and peripheral tumor specimens. Correlation between larger tumor specimen and increasing core biopsy number for CD8+ lymphocytes (**a**) peripheral core, (**b**) central core biopsies and CD68+ macrophages, (**c**) peripheral core, and (**d**) central core biopsies. Correlation differences between central and peripheral cores for (**e**) lymphocytes and (**f**) macrophages. P-values for Wilcoxon RankSum test are shown.

We next determined the optimal number of small biopsies needed. We focused on a subset of 11 patients for whom four central and four peripheral biopsies were available for analysis (Fig. 4). To accurately observe the variability in values, we computed and generated a distribution of Pearson correlation coefficients R between cell count values in larger specimens versus cell count values in an increasing number of core biopsies (separately from central and peripheral areas of the tumor) (Fig. 5). The resultant distribution consisted of correlations between the slides of the larger tumor specimen and of 50 randomly drawn and repeating combinations of biopsy cores of the specific type. Additionally, we calculated non-adjusted ICC with 95% CI, where ICC values less than 0.5 are indicative of poor concordance, 0.5 to 0.75 indicated moderate concordance, and 0.75 to 0.9 indicate good concordance, and values greater than 0.90 indicate excellent concordance^15^. We found that in both central and peripheral cores, the correlation between CD8+ cell densities in larger tumor specimens compared with those in small biopsies was strong when two biopsies were used (Fig. 5a,b).

Although increasing the number of core biopsies led to increased statistical significance, only two biopsies were needed for a strong correlation to larger tumor specimens (R > 0.7). This was replicated by non-adjusted ICC analysis, where two central biopsies and two peripheral biopsies yielded a moderate concordance with larger tumor specimen (ICC [CI range] central 0.61 [0.05–0.89]; peripheral 0.77 [0.33–0.94]) (Supplementary Table S2). Although central and peripheral cores were found to be significantly different (*P* = 0.03), the overall correlation to larger tumor remained strong, with R > 0.7 for both sets (Fig. 5e).

Correlation between CD68+ stained cells in the larger tumor specimen compared with small biopsies was met when two central or two peripheral biopsies were used (Fig. 5c,d). Finally, we compared CD68+ cell densities in central *versus* peripheral biopsies; although there was a higher correlation between peripheral biopsies and larger tumor, both biopsy sites yielded a moderate correlation to larger tumor (R = 0.3 to 0.7), regardless of location (central versus peripheral) (Fig. 5f). ICC analysis revealed poor concordance when comparing CD68 counts between larger tumor and tumor core biopsies (Supplementary Table S2). Thus, we concluded that the number of biopsies to be taken was dependent on the marker assessed. However, when comparing all markers, a total of two biopsies taken either centrally or peripherally yielded a moderate to strong correlation with immune populations in HGSC larger tumor.

## Discussion

Our ability to use components of the TME for therapeutic and prognostic strategies requires a more complete understanding of the complexities of the TME. Sufficient sampling of the tumor may offer insights into the diverse and complex interactions between immune, tumor, and stromal cells. Here, we have established a methodology to evaluate the TME components, providing a high-throughput protocol for clinical translation. This method benefits from the bioinformatics power of inForm Cell analysis and the use of multiplex IHC staining to identify differing cell populations. The use of multiplex staining is important since it allows for identification of specific individual cell populations in one tissue specimen.

An increasing number of clinical trials require submission of tissue specimens, either from archived specimens or fresh biopsies taken from patients. These tissue specimens help to identify biomarkers for enrollment in trials or are saved for monitoring and correlative studies. Often enrollment in clinical trials can be delayed considerably because of the requirement to have a research biopsy. For instance, patients with advanced non–small cell lung cancer who enrolled in clinical trials received treatment one week earlier in trials that did not have a mandatory tissue sample requirement^16^. In addition, almost 30% of patients had insufficient tissue on the biopsy specimen for analysis^16^. Patient reluctance to enter clinical trials for which tissue biopsy is a requirement highlights the importance of establishing standardization of protocols in order to use the limited amount of tissue in an efficient and timely fashion. Circulating tumor DNA may be used broadly as a tool for analysis of disease burden and genomic analyses, but it has limited utility for assessing the TME or tumor heterogeneity^17^.

Tissue biopsy samples are not only important for use in clinical trial eligibility and monitoring, but also may allow clinicians to determine potential responders versus non-responders before treatment. For instance, the ability to quantify T-cell infiltration may predict checkpoint blockade responsiveness^18,19^, and vessel density has been associated with greater benefit from bevacizumab in some studies^20^.

Several studies have focused on improving biopsy quality to yield sufficient material through protocol-specific and evidence-based practice guidelines; however, not much emphasis has been placed on determining whether these biopsies are truly representative of the larger tumor^21,22^. Currently, there are no established guidelines for a maximum number of core specimens, and those participating in clinical trials may undergo three to six core specimens per procedure^23^. We found out that the evaluation of tumor-associated stroma needs a minimum of two core biopsies, regardless of location, to be faithfully represented. Although the amount of tumor-associated stroma differed, depending on whether peripheral or central areas were analyzed, tumor epithelial areas were evenly distributed among peripheral and central cores. This might signify that tumor-associated stroma heterogeneity requires a larger number of analyzed sample areas in order to be fully represented, whereas tumor epithelial areas seem to be more homogeneous throughout the tissue specimen.

Limitations of our study include small sample size. Due to sample size limitations and the exploratory nature of our study, we used Pearson’s correlation and non-adjusted ICC to assess concordance between larger tumor specimen and core biopsy. Future studies could use Spearman correlation and adjusted ICC provided they have adequate sample size for each site. We also did not assess how concordance between larger tumor and core biopsy varies between tumor site (primary versus metastatic), which is a question we would like to expand on in future studies.

As the number of clinical trials increases and the development of markers predictive of therapy response expands, there will be a growing need for adequate tissue specimens. Our analyses suggest that a small tissue biopsy can adequately inform clinicians of specific components of the TME. We found that a number of positively stained CD8 cells could be reliably represented by two tissue biopsies regardless of location. CD68+ cells were adequately represented by two tissue biopsies as well, with a higher percentage of cells found in peripheral cores, which correlated well with the larger tissue specimen. We found that PD-L1 expression was poorly represented by tissue biopsies in our cohort of HGSC patients; thus, clinicians and researchers should be aware of the limitations that small tissue biopsies may pose for evaluating some checkpoints. Collectively, this study offers new insight into the reliability of tumor microarrays and reveals the limitations in assessing tumor specimens in a high-throughput fashion.

## Supplementary information

Supplementary Appendix

## Data Availability

The datasets generated during and/or analyzed during the current study are available from the corresponding author on request.
