# Supplementary material for: Tumor core biopsies adequately represent immune microenvironment of high-grade serous carcinoma
Source: Sci Rep. 2019 Nov 26;9:17589. doi: 10.1038/s41598-019-53872-1 (PMC6879510; doi:10.1038/s41598-019-53872-1)
Supplement: Supplementary file 1 — Supplementary Appendix [file 41598_2019_53872_MOESM1_ESM.docx]

**Tumor core biopsies adequately represent immune microenvironment of high-grade serous carcinoma**

**Authors:** Olivia D. Lara^1^, Santhoshi Krishnan^2,3^, Zhihui Wang^4^, Sara Corvigno^1^, YanPing Zhong^5,6^, Yasmin Lyons^1^, Robert Dood^1^, Wei Hu^1^, Lisha Qi^5^, Jinsong Liu^5^, Robert L. Coleman^1^, Shannon N. Westin^1^, Nicole D. Fleming^1^, Vittorio Cristini^4,7^, Arvind Rao^2,3,8^, Jared Burks^9^ & Anil K. Sood^1,10 ,*^

^1^Department of Gynecologic Oncology and Reproductive Medicine, The University of Texas MD Anderson Cancer Center, Houston, TX 77030, USA

^2^Department of Electrical and Computer Engineering, Rice University, Houston TX 77030, USA

^3^ Department of Computational Medicine and Bioinformatics, University of Michigan, Ann Arbor, MI 48109, USA

^4^Mathematics in Medicine Program, Houston Methodist Research Institute, Houston, TX 77030, USA

^5^Department of Pathology, The University of Texas MD Anderson Cancer Center, Houston, TX 77030, USA

^6^ Department of Pathology, The First Hospital of Jilin University, Changchun, China

^7^Department of Imaging Physics, The University of Texas MD Anderson Cancer Center, Houston, TX 77030, USA

^8^Department of Radiation Oncology, University of Michigan, Ann Arbor, MI 48109, USA

^9^Flow Cytometry and Cell Imaging, The University of Texas MD Anderson Cancer Center, Houston, TX 77030, USA

^10^Center for RNA Interference and Non-Coding RNA, The University of Texas MD Anderson Cancer Center, Houston, TX 77030, USA

^*^Corresponding Author: Anil K. Sood, MD. The University of Texas MD Anderson Cancer Center, 1515 Holcombe Blvd, unit 1362, Houston, Texas, 77030. [asood@mdanderson.org](mailto:asood@mdanderson.org)

**Supplementary Information**


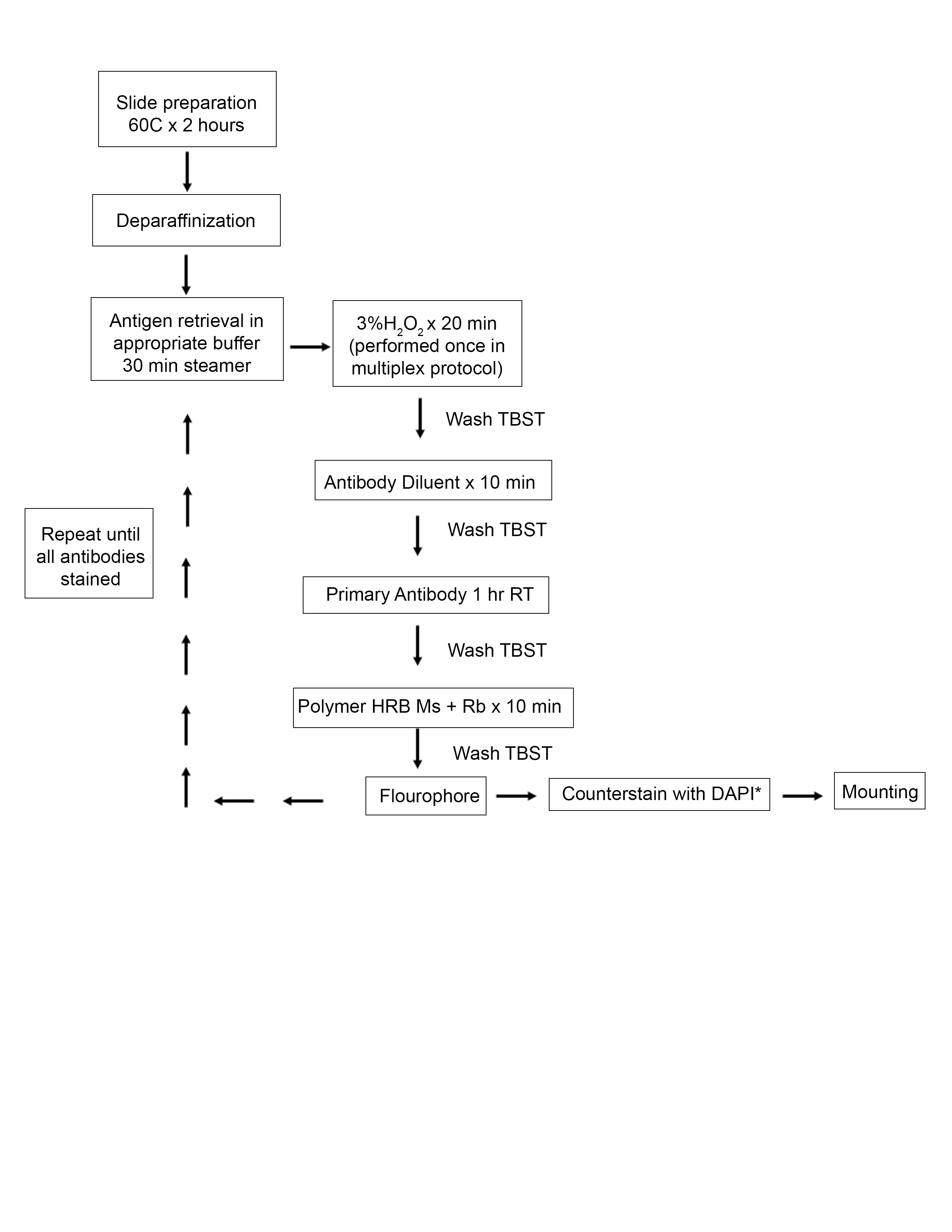


**Supplementary Figure S1. Opal multiplex staining workflow.** Steps of the multiplex protocol starting with deparaffinization and ending with mounting. RT: Room temperature, * counterstain with DAPI after staining will all fluorophores.


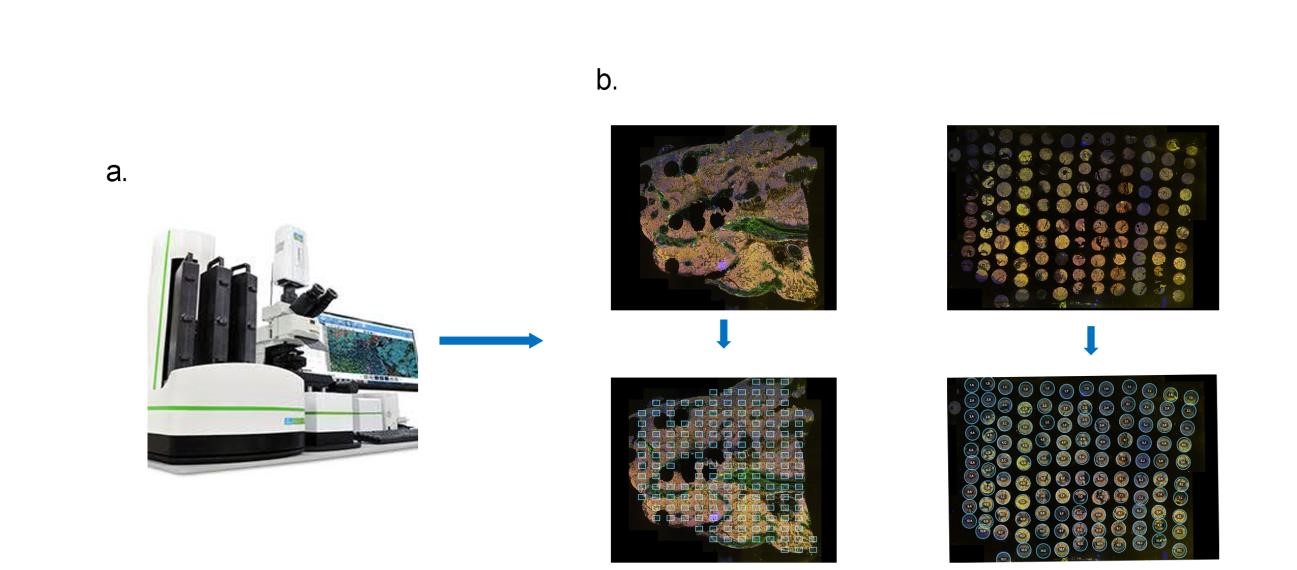


**Supplementary Figure S2. Image acquisition work flow.** (**a**) Vectra-automated Imaging System used to take low-powered images for each tumor tissue slide and tissue microarray. Image acquired from [https://www.akoyabio.com/phenopticstm/instruments/vectra-30-automated-quantitative-pathology-imaging-system-6-slide](https://urldefense.proofpoint.com/v2/url?u=https-3A__www.akoyabio.com_phenopticstm_instruments_vectra-2D30-2Dautomated-2Dquantitative-2Dpathology-2Dimaging-2Dsystem-2D6-2Dslide&d=DwMFaQ&c=j5oPpO0eBH1iio48DtsedeElZfc04rx3ExJHeIIZuCs&r=oI3TvIOjSPucewR56yZLDu0_tEoIV_m7SD5LtEOSaoQ&m=dJDDnAwTZfBbr1MqUBY8OE0iG2ibTjyh6OvX7Y-lxSY&s=e1xs2CitNvV30pmIWwauWFXUFIxT8B3zwKgRbXBzT6g&e=) (**b**) Phenochart whole-slide viewer used to annotate slides and acquire representative images of entire tumor specimen. One image was taken per core.


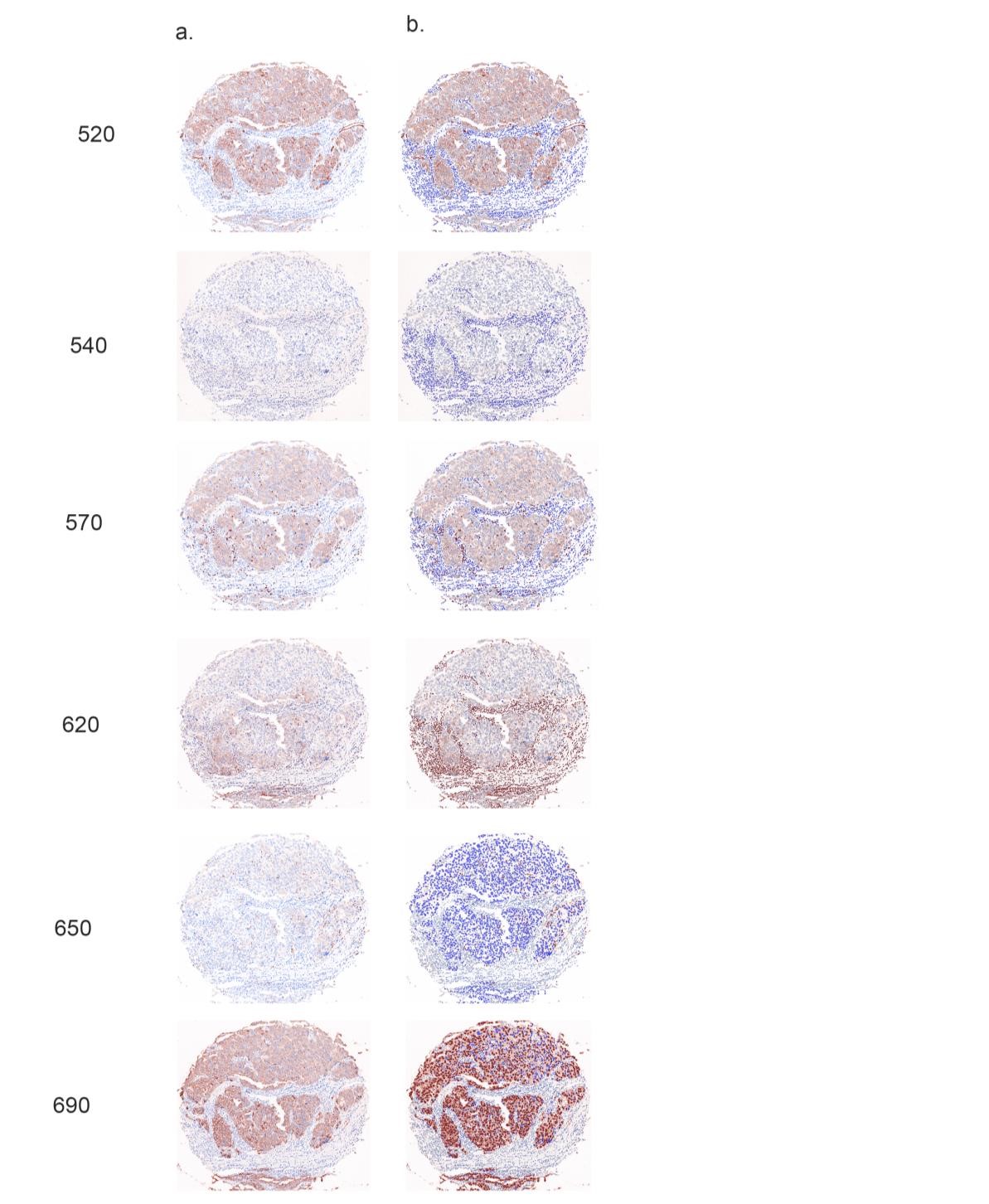


**Supplementary Figure S3. Spectral unmixing.** (**a**) Each multiplex image was spectrally unmixed to identify individual fluorophores. (**b**) Cell segmentation then separated cells on a cell-by-cell basis. Thresholds were set to identify only those deeply positive, represented by brown nuclei (virtual DAB staining).


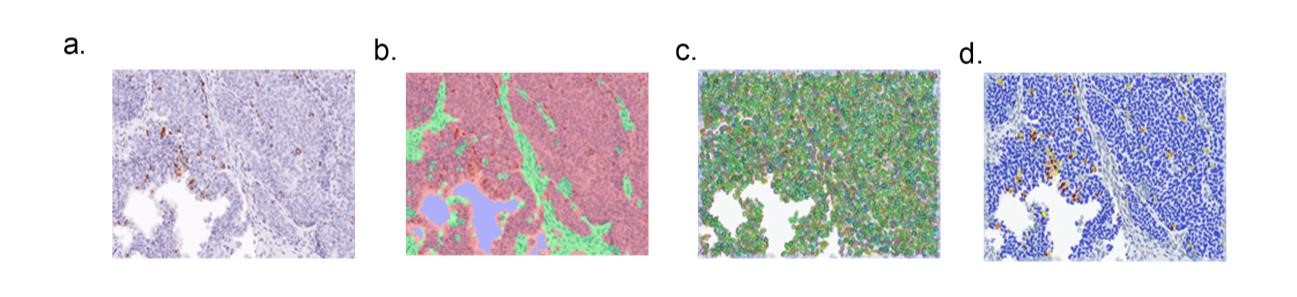


**Supplementary Figure S4. Tissue and cellular segmentation.** (**a**) Unsegmented slide. InForm Analysis used for (**b**) tissue segmentation, in which interface was trained to identify tumor (red), stroma (green), non-tissue (blue), and fat (yellow). Interface was created with representative images for each tumor specimen and then algorithm was performed in batch analysis for cohort of slides. The percentage of stromal tissue and tumor tissue was accurately estimated by using inForm Analysis interface. (**c**) InForm Analysis was used for cell-by-cell analysis. Interface was created with representative images for each tumor specimen, and then an algorithm was performed in batch analysis for cohort of slides. Correlation between total positive staining cells for each core was compared with the average number of positive cells in representative tumor sample. (**d**) Positively staining cells identified with inForm analysis with thresholds were set for low to highly positive cells based on intensity of stain. The number of positively staining cells was quantified.


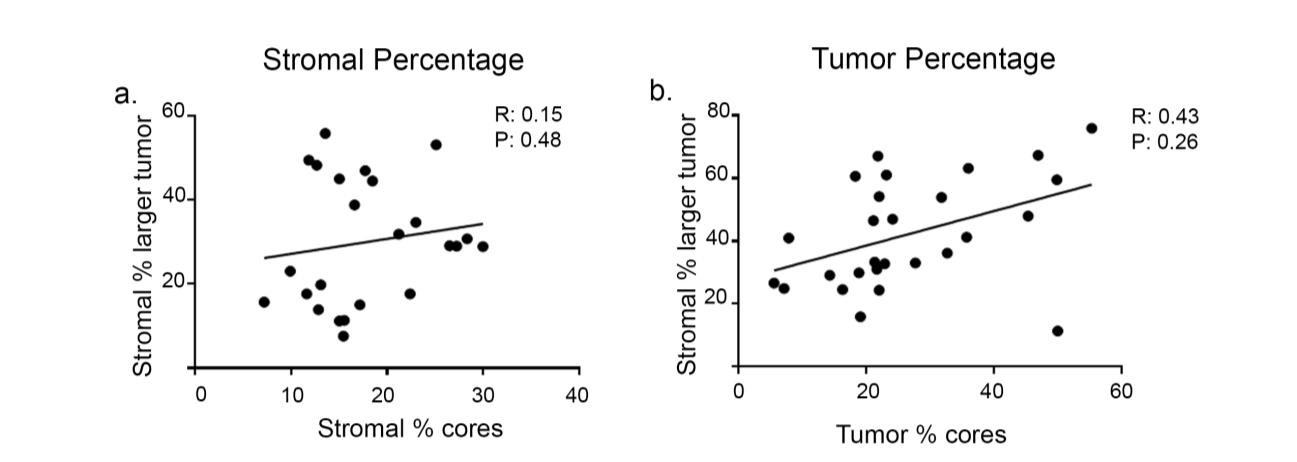


**Supplementary Figure S5. Correlation between stroma and tumor epithelial areas in all patients.** Correlation of stromal (**a**) and tumor (**b**) percentages between larger tumor and all core biopsies for 26 patients. Pearson correlation coefficient (*R*) and *P* values for each correlation analysis are shown in insets.


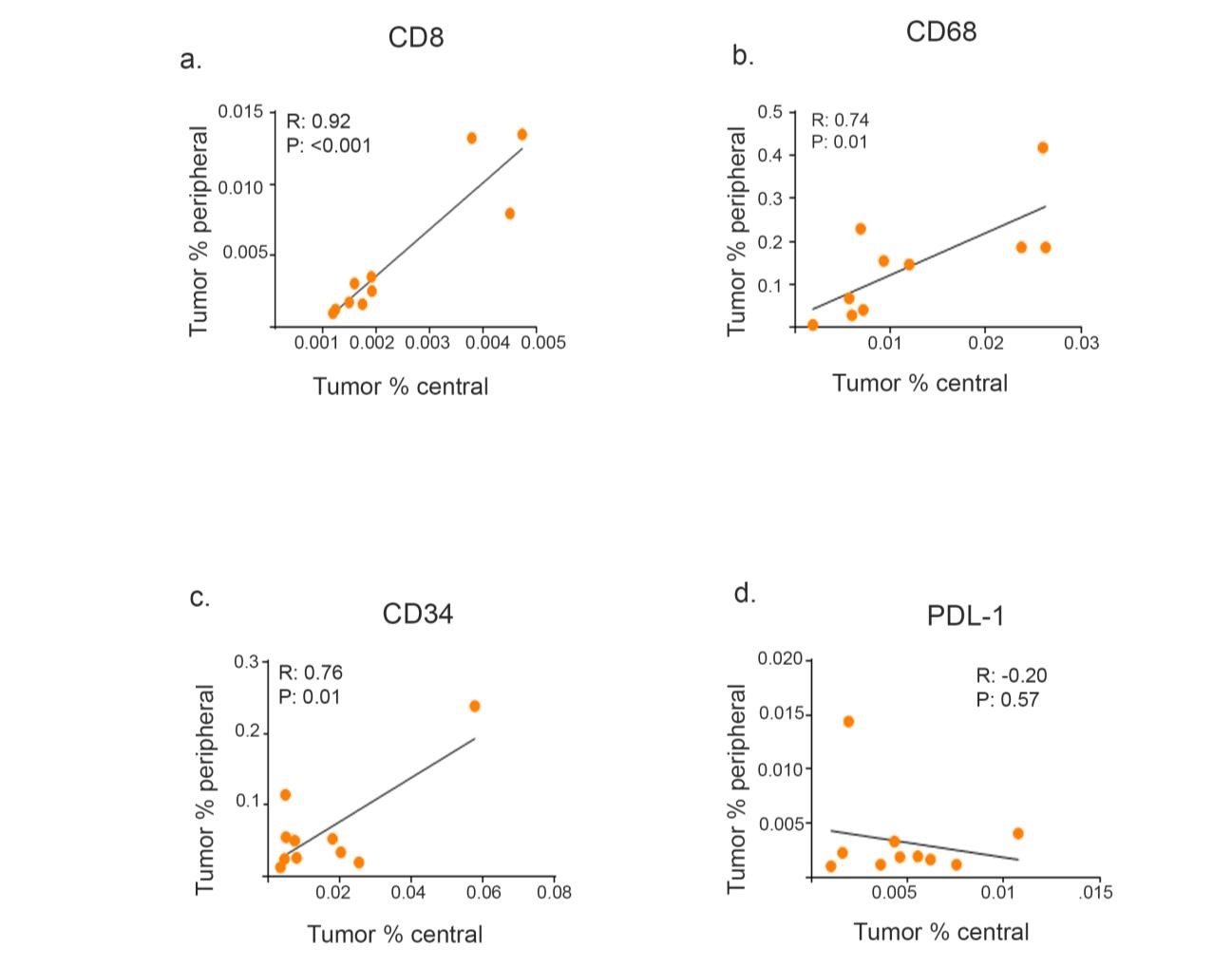


**Supplementary Figure S6. Central and peripheral core correlation.** Correlation between four central core biopsies and four peripheral core biopsies for CD8 (**a**), CD68 (**b**), CD34 (**c**), and PDL1 (**d**).

**Supplementary Table S1.** Opal multiple staining reagents

|  | Antibody | |  |  | Fluorophore | | |  |
| --- | --- | --- | --- | --- | --- | --- | --- | --- |
| Antigen | Concentration | Brand | Catalog No. | Antigen Retrieval Buffer | Opal | Brand | Catalog No. | TSA Concentration |
| CD34 | 1:75 | DAKO | M716501 | AR 9 | 520 | Perkin Elmer | FP1487A | 1:75 |
| PDL1 | 1:150 | Cell Signal | 13684D | AR 9 | 540 | Perkin Elmer | FP1494A | 1:50 |
| CD8 | 1:30 | DAKO | M710301 | AR 9 | 570 | Perkin Elmer | FP1488A | 1:50 |
| CD68 | 1:40 | DAKO | M087601 | AR 6 | 650 | Perkin Elmer | FP1496A | 1:150 |
| Fibroblast Activated protein | 1:250 | abcam | Ab207178 | AR 9 | 620 | Perkin Elmer | FP1495A | 1:75 |
| Cytokeratin | 1:50 | DAKO | M3515 | AR 9 | 690 | Perkin Elmer | FP1497A | 1:100 |

**Supplementary Table S2.** Inter-class correlation and 95% CI for CD8 and CD68

| Marker | Large Slide Image vs Core Number Comparison | Intra-class Correlation | | | F-test | | | |
| --- | --- | --- | --- | --- | --- | --- | --- | --- |
|  |  | Value | Lower Bound(CI) | Upper Bound(CI) | Value | Df1 | df2 | P-value |
| CD8 | 2-Central Core | 0.614 | 0.057 | 0.886 | 5.3 | 9 | 7.53 | 0.0162 |
|  | 3-Central Core | 0.537 | -0.021 | 0.855 | 4.1 | 9 | 8.07 | 0.0293 |
|  | 4-Central Core | 0.574 | 0.016 | 0.87 | 4.62 | 9 | 7.81 | 0.0222 |
|  | 2-Peripheral Core | 0.775 | 0.334 | 0.939 | 7.53 | 9 | 9.67 | 0.0023 |
|  | 3-Peripheral Core | 0.755 | 0.299 | 0.932 | 697 | 9 | 9.83 | 0.00293 |
|  | 4-Peripheral Core | 0.752 | 0.29 | 0.932 | 6.83 | 9 | 9.77 | 0.00323 |
| CD68 | 2-Central Core | 0.0621 | -0.093 | 0.403 | 1.51 | 9 | 7.77 | 0.288 |
|  | 3-Central Core | 0.0881 | -0.094 | 0.455 | 1.75 | 9 | 6.31 | 0.249 |
|  | 4-Central Core | 0.0749 | -0.09 | 0.426 | 1.65 | 9 | 6.8 | 0.264 |
|  | 2-Peripheral Core | 0.294 | -0.246 | 0.743 | 2 | 9 | 9.93 | 0.148 |
|  | 3-Peripheral Core | 0.314 | -0.241 | 0.755 | 2.07 | 9 | 9.96 | 0.137 |
|  | 4-Peripheral Core | 0.329 | -0.237 | 0.764 | 2.12 | 9 | 9.98 | 0.128 |
